# Sarcomatoid renal cell carcinoma masquerading as xanthogranulomatous pyelonephritis: a case report

**DOI:** 10.3389/fonc.2026.1798397

**Published:** 2026-07-22

**Authors:** Chenzhao Hua, Tingting Li, Xianqiao Li, Shuhua Duan

**Affiliations:** 1 Department of Urology, Ward One, Shengli Oilfield Central Hospital, Dongying, Shandong, China; 2 Department of Urology, Ward Two, Shengli Oilfield Central Hospital, Dongying, Shandong, China; 3 Department of Nephrology, Shengli Oilfield Central Hospital, Dongying, Shandong, China; 4 Department of Medical Imaging, Shengli Oilfield Central Hospital, Dongying, Shandong, China

**Keywords:** diagnostic challenge, early surgical intervention, misdiagnosis, radiological red flags, renal cell carcinoma, sarcomatoid renal cell carcinoma, xanthogranulomatous pyelonephritis

## Abstract

**Background:**

Sarcomatoid renal cell carcinoma (sRCC) is a rare, highly aggressive malignancy that can closely mimic xanthogranulomatous pyelonephritis (XGP) on clinical, laboratory, and radiological grounds. While sRCC masquerading as XGP has been previously reported, existing literature lacks a structured analysis of the specific features that discriminate these entities and a practical framework for clinical decision-making.

**Case presentation:**

A 65-year-old female presented with a four-month history of anorexia and one week of postprandial abdominal distension. Contrast-enhanced CT demonstrated right renal enlargement with heterogeneous density, nephrolithiasis, and a bear-paw-like appearance, leading to an initial diagnosis of XGP. However, several features were inconsistent with typical XGP: persistently negative urine and blood cultures, an irregular solid component with heterogeneous arterial-phase enhancement, subtle renal vein irregularity suggestive of vascular invasion, progressive transfusion-requiring anemia, and persistent high fever despite 24 days of escalating broad-spectrum antibiotics (culminating in meropenem plus vancomycin). Repeat CT showed no radiological improvement. Right radical nephrectomy was performed on hospital day 31. Histopathology confirmed sRCC with lymphovascular invasion and a Ki-67 index of 50–55%. Despite initial recovery, the patient developed rapid multi-organ metastasis and succumbed to the disease.

**Conclusion:**

When a presumed renal infection is culture-negative, antibiotic-refractory, associated with progressive anemia, and radiologically non-resolving, malignancy should be suspected early. The presence of an irregular solid enhancing component and vascular involvement on CT are malignancy-specific red flags that should not be overlooked. A bear-paw-like appearance does not exclude RCC. Short-interval failure of antibiotic therapy (7–10 days) should trigger surgical exploration or tissue diagnosis rather than prolonged empirical treatment.

## Introduction

Xanthogranulomatous pyelonephritis (XGP) is a rare but well-recognized chronic destructive inflammatory condition of the kidney, typically associated with nephrolithiasis, urinary tract obstruction, and chronic bacterial infection (1). Sarcomatoid renal cell carcinoma (sRCC), by contrast, is a highly aggressive dedifferentiated variant of renal cell carcinoma (RCC) accounting for approximately 5% of all RCC cases, with a median overall survival of less than 12 months from diagnosis (2). Despite their fundamentally different etiologies, sRCC and XGP share a striking clinical and radiological resemblance: both can present with constitutional symptoms, leukocytosis, renal enlargement, intralesional necrosis, and nephrolithiasis, rendering their differentiation a formidable diagnostic challenge (1, 2).

Although cases of sRCC mimicking XGP have been reported in the literature, the existing reports have generally lacked a systematic analysis of discriminating features or actionable clinical guidance regarding the optimal timing of surgical intervention (3–5). Critically, the question of when to abandon antibiotic therapy and proceed to surgical exploration has not been addressed with sufficient specificity.

We present a case of sRCC initially managed as XGP for 24 days and provide a structured analysis of the specific clinical, laboratory, and radiological red flags that—if recognized earlier—should have prompted earlier consideration of malignancy. The central learning point of this case is that a renal inflammatory-appearing lesion that does not respond to infection-directed therapy should be treated as a potential malignancy until proven otherwise by histopathology.

## Case presentation

### Phase 1: initial presentation and diagnosis of presumptive XGP (days 1–3)

A 65-year-old Han Chinese female, retired, living with her spouse, and functionally independent prior to this illness, presented to the gastroenterology outpatient clinic on Day 1 (January 4, 2019) with a four-month history of progressive anorexia and a one-week history of postprandial abdominal distension. She reported an unintentional weight loss of approximately 4 kg over the preceding three months. She denied dysuria, urinary frequency, frank hematuria, flank pain, or rigors at the time of initial presentation.

Her medical history was significant for hypertension of 10 years’ duration, managed with oral nifedipine 30 mg once daily (blood pressure controlled at approximately 120/80 mmHg). She had no prior history of urological disease, renal calculi, urinary tract infections, or prior urological interventions. She had no known history of diabetes mellitus, immunosuppression, or chronic corticosteroid use. She was a lifelong non-smoker and denied alcohol or illicit substance use.

Family and psychosocial history: The patient denied any family history of hereditary renal disease, urological malignancy, or other hereditary conditions. No first-degree relatives were known to have been diagnosed with RCC or other urological cancers. From a psychosocial perspective, the patient was cognitively intact, emotionally supported by her family, and resided in an urban environment with adequate access to healthcare.

Relevant past interventions: Gastroscopy and colonoscopy performed at an external facility three to four months prior to this admission had excluded gastrointestinal malignancy, and gastrointestinal tumor markers (CEA, CA19-9, AFP) were within normal limits at that time. No prior renal imaging had been performed. The patient had not received any antibiotics, immunosuppressants, or other relevant medications prior to this admission.

On admission, physical examination revealed right costovertebral angle tenderness. Laboratory testing showed leukocytosis (WBC 14.7 × 10^9^/L with neutrophilia), anemia (hemoglobin 84 g/L), marked pyuria (urinary WBC 1904/µL), hematuria (urinary RBC 121/µL), and urine dipstick leukocyte esterase 3 +. Urine and blood cultures remained repeatedly negative despite ongoing pyuria and fever.

**Figure 1:**
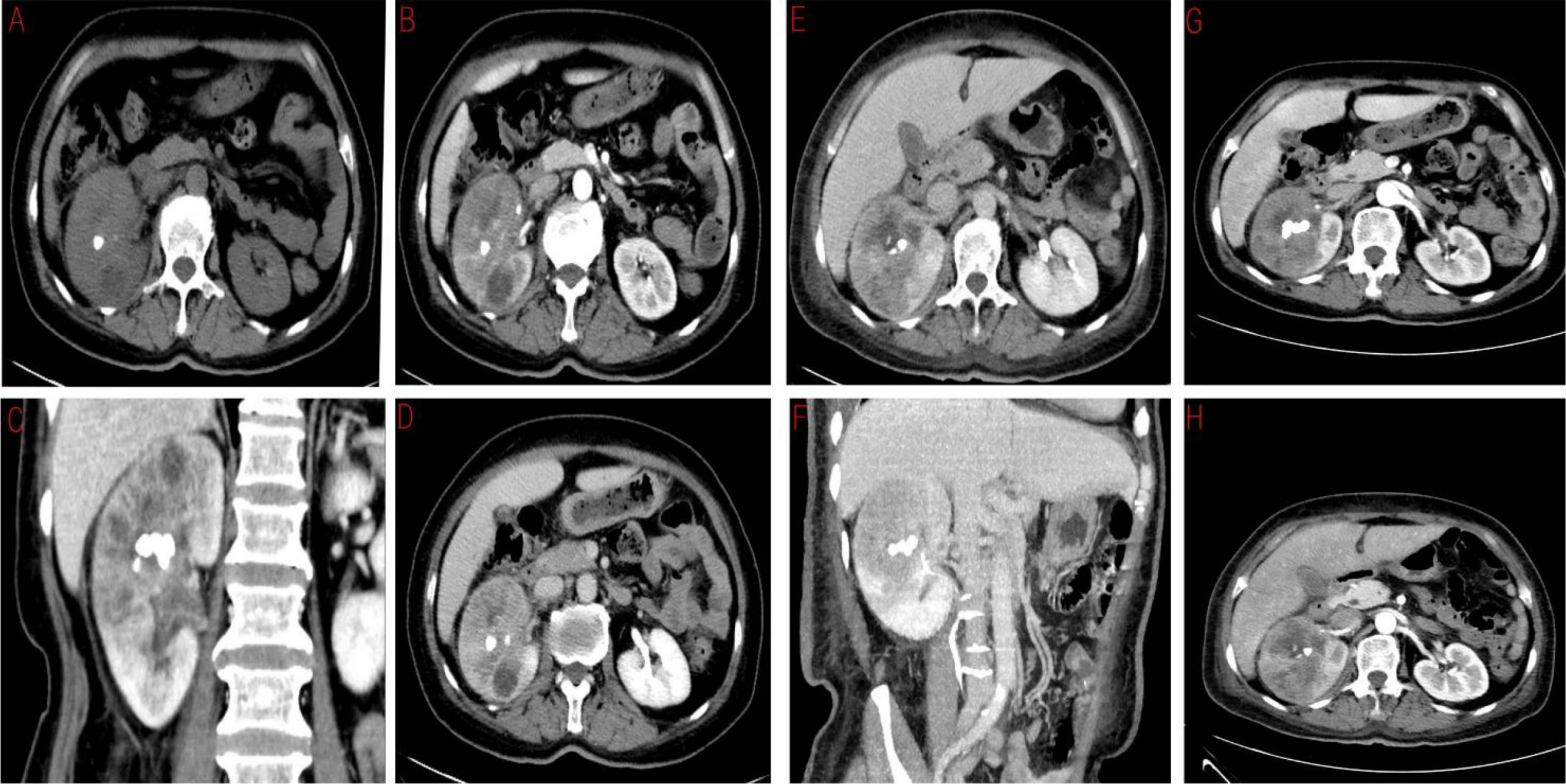
**(A)** Non-contrast CT scan demonstrating right renal enlargement and nephrolithiasis. **(B)** Arterial phase contrast-enhanced CT showing heterogeneous enhancement. **(C)** Venous phase contrast-enhanced CT. **(D)** Delayed phase contrast-enhanced CT. **(E)** Bear’s paw sign. **(F)** The renal veins are slightly irregular. **(G)** First enhanced CT (Day 4). **(H)** Second enhanced CT(Day 27).

Non-contrast CT performed on Day 2 (January 5, 2019), followed by contrast-enhanced CT on Day 4(January 7, 2019) (**Figure 1**), demonstrated right renal enlargement with heterogeneous parenchymal density, associated nephrolithiasis, and perinephric inflammatory stranding. These features resemble bear’s paw signs (**Figure 1E**) and contribute to the primary radiological diagnosis of right XGP.

### Phase 2: antibiotic therapy and emergence of red flags (days 4–24)

Despite the radiological impression, several features at this stage were atypical for XGP and should have prompted earlier reassessment (**Table 1**):

**Table 1:** Comparative features of XGP vs. sRCC in this case.

Feature	Typical XGP	sRCC	This case
Urine/blood cultures	Positive (E. coli, Proteus)	Negative	Negative
“Bear paw sign” on CT	Present (classic)	Absent	Atypical
Solid enhancing component	Absent or minimal	Irregular, heterogeneous	Present
Vascular invasion on CT	Absent	May be present	Present
Response to antibiotics	Partial improvement	No improvement	No improvement
Progressive anemia	Uncommon	Common	Progressive
Radiological change with treatment	Improvement expected	No change or progression	No change
Culture-negative pyuria	Atypical	Common (sterile inflammation)	Present

1. Persistently negative urine and blood cultures(multiple samples obtained): Typical bacterial XGP is associated with positive cultures in the majority of cases (most commonly Escherichia coli or Proteus mirabilis). Repeatedly negative cultures in the setting of florid pyuria are inconsistent with classical XGP and should raise suspicion for a non-infectious etiology or tumor-induced sterile inflammation.
2. Presence of an irregular solid enhancing component: In retrospect, the CT images demonstrated an ill-defined solid component with heterogeneous arterial phase enhancement (**Figure 1B**) that is more consistent with malignancy than with pure inflammatory disease. Irregular solid components within a renal mass should always raise concern for malignancy.
3. Radiological features suggestive of vascular invasion: Subtle irregularity of the renal vein on venous phase imaging (**Figure 1F**) was noted in retrospect, a feature that strongly favors RCC over XGP.

Antibiotic therapy was initiated on Day 4 following the radiological diagnosis of presumptive XGP. The initial choice of oral amoxicillin-clavulanate (625 mg three times daily) was empirically selected based on the presumed diagnosis of XGP caused by common Gram-negative uropathogens. Given the absence of clinical improvement and persistent fever after 48 hours, therapy was escalated to intravenous piperacillin-tazobactam (4.5 g every 8 hours) on Day 6, targeting a broader Gram-negative spectrum including *Pseudomonas aeruginosa*. When fever and leukocytosis persisted despite 72 hours of piperacillin-tazobactam, therapy was further escalated to intravenous Cefoperazone/Sulbactam on Day 10 to provide enhanced beta-lactamase coverage. Failure to achieve clinical defervescence or laboratory improvement by Day 14 prompted escalation to meropenem (1 g every 8 hours) combined with vancomycin (15 mg/kg every 12 hours, with renal dose adjustment), targeting carbapenem-resistant organisms and Gram-positive pathogens including methicillin-resistant *Staphylococcus aureus* (MRSA).

Over this 24-day period, the patient exhibited:

Persistent high fever (recurrent temperature spikes >38.5 °C) despite broad-spectrum coverage:

Progressive anemia: hemoglobin declined from 84 g/L at admission to 74 g/L by Day 16 (January 19, 2019) and 69 g/L by Day 19 (January 22, 2019), necessitating repeated packed red blood cell transfusions.

No radiological improvement: repeat contrast-enhanced CT on Day 27 (January 30, 2019) showed right kidney enlargement with heterogeneous density without significant interval change compared to the initial scan.

### Phase 3: surgical decision and operative management (days 28–31)

Following multidisciplinary consultation involving urology, nephrology, and radiology, a right radical nephrectomy was recommended for diagnostic clarification and definitive management. On Day 31 (February 3, 2019), the patient underwent right radical nephrectomy under general anesthesia with an estimated intraoperative blood loss of 500 mL. Due to hemodynamic instability, the patient was transferred to the Intensive Care Unit (ICU) post-operatively and stepped down to the urology ward on post-operative day 2 (February 5, 2019). The patient became afebrile following surgery, with subsequent normalization of body temperature.

### Phase 4: pathological diagnosis and outcome (post-operative)

Post-operative histopathological examination results confirmed the diagnosis of right renal cell carcinoma, sarcomatoid type (sRCC). Key pathological findings included:

Gross examination: Tumor measuring 9.5 × 6.0 × 4.0 cm with extensive central necrosis and an intralesional renal calculus.

Microscopic examination: Invasion of the renal capsule, renal pelvis, and perinephric fat; lymphovascular invasion present; perineural invasion absent; ureteral and vascular surgical margins negative.

**Figure 2:**
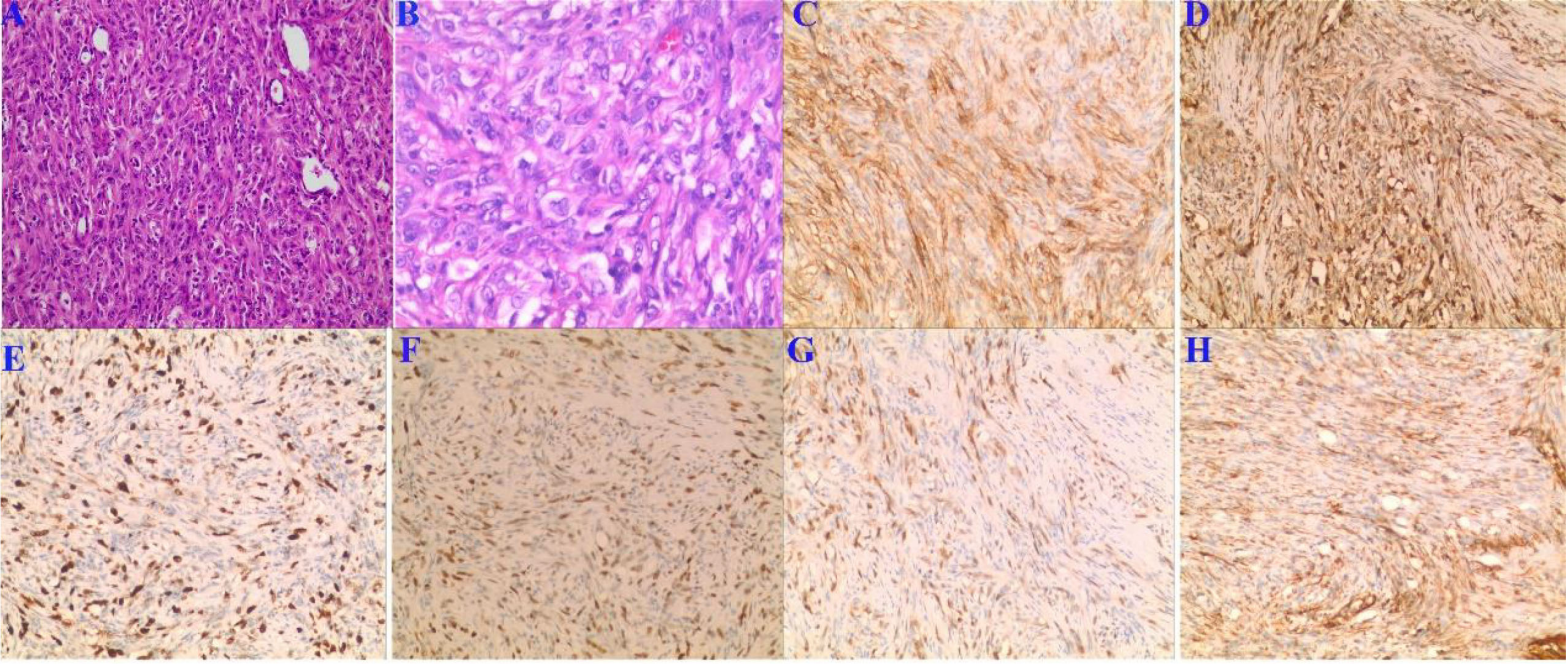
Histopathological and Immunohistochemical Findings. **(A)** Hematoxylin and Eosin (H&E) stain, 100x magnification, showing features of sRCC. **(B)** H&E stain, 200x magnification, detailing cellular atypia. **(C)** CD10 positivity. **(D)** CD68 positivity. **(E)** Ki-67 staining demonstrating high proliferation index. **(F)** p53 positivity. **(G)** PAX-8 positivity. **(H)** Vimentin positivity.

Immunohistochemistry (IHC): Strong positivity (3+) for CKpan and PAX-8 (confirming renal epithelial origin); moderate positivity (2+) for Vimentin, CD10, CD68, and p53; Ki-67 proliferation index 50–55% (**Figure 2**, **Table 2**).

**Table 2:** Summary of important auxiliary investigations and results.

Category	Examination/test	Key findings
Laboratory findings		
WBC	Day 1	14.7 x 10^9^/L,
Hemoglobin	Day 1	84 g/L,
	Day 16	74 g/L
	Day 19	69 g/L
	Post-operative	143 g/L
Urinalysis	Day 1	RBC 121/µL, WBC 1904/µL
	Day 7	RBC 664/µL, WBC 899/µL
	Urine cultures (multiple)	Negative
Imaging findings		
CT	Abdomen Contrast Enhanced (Day 4)	Findings suggestive of right kidney inflammatory lesion (XGP)?;
	Abdomen Contrast Enhanced (Repeat, Day 27)	Right kidney enlarged, heterogeneous density; No significant change from previous scan.
Pathology findings		
Gross examination	Size	9.5×6.0×4.0 cm
Microscopic examination	Features	Extensive central necrosis; Renal calculus present.
	Invasion	Renal capsule, renal pelvis, perirenal fat; Lymphovascular invasion present.
	Margins	Negative.
Immunohistochemistry	Key markers	Positive: CKpan (3+), PAX-8(3+), Vimentin (2+), CD10(2+), CD68(2+), p53(2+).
	Proliferation index	Ki-67: 50-55%

The high Ki-67 index and lymphovascular invasion confirmed the highly aggressive biological behavior of this tumor. Post-operatively, the patient’s hemoglobin recovered to 143 g/L and pyuria resolved. However, post-discharge follow-up revealed rapid disease progression with the development of multi-organ metastases, and the patient ultimately succumbed to her disease within months of surgery.

**Figure 3:**
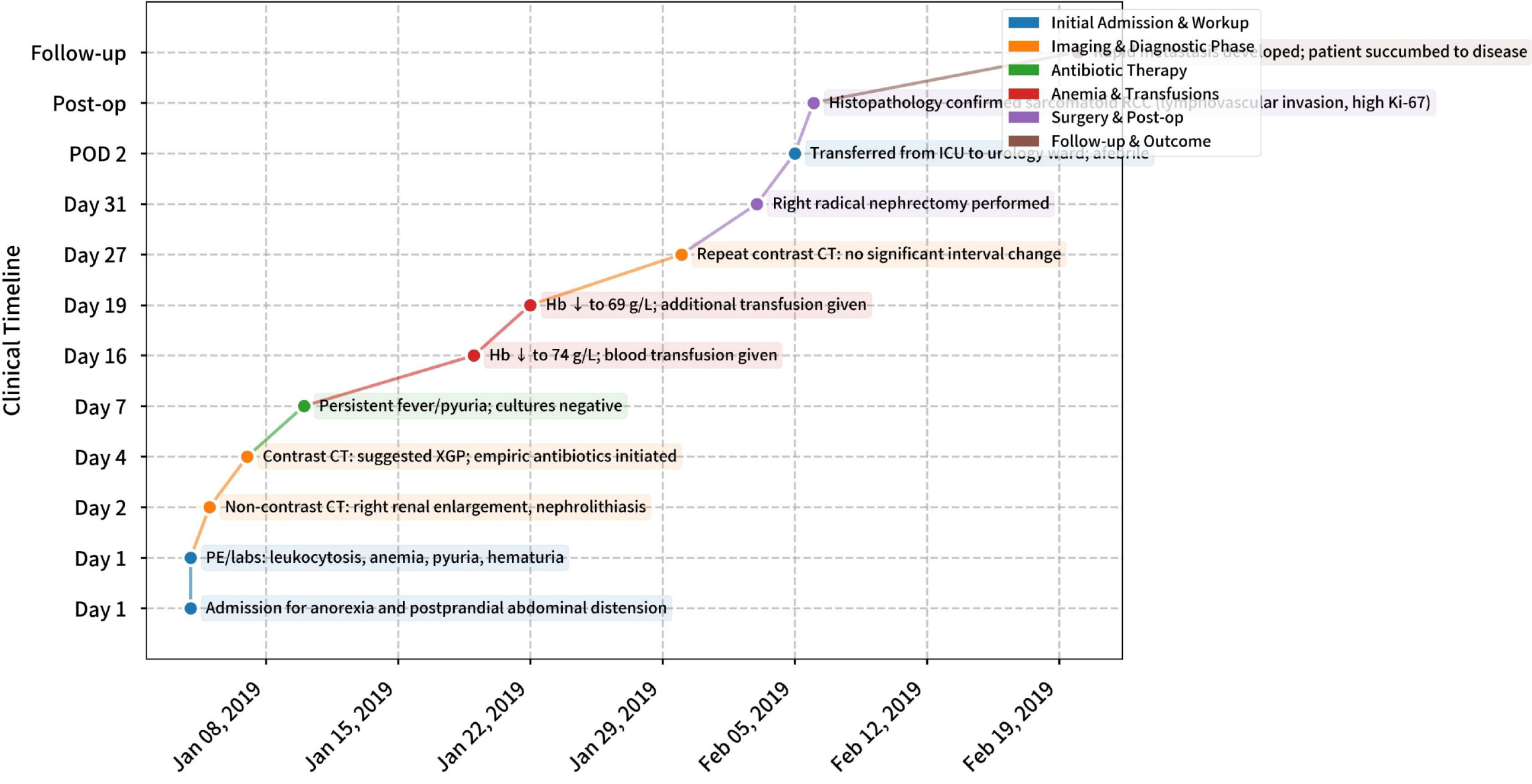
Clinical course timeline: sarcomatoid renal cell carcinoma.

The patient’s clinical course, including major events, laboratory findings, and imaging results, is detailed chronologically in **Table 2** and illustrated in **Figure 3**.

## Discussion

Distinguishing sRCC from XGP is a formidable clinical challenge due to their overlapping presentations. Both conditions can manifest with constitutional symptoms, leukocytosis, and renal masses containing calculi and necrosis (1, 6, 7). As illustrated in this case, these similarities often lead to an initial presumption of XGP, potentially delaying life-saving oncologic intervention.

XGP, a severe chronic inflammatory process, typically necessitates anti-infective therapy and/or nephrectomy and generally carries a relatively favorable prognosis (8). In stark contrast, sRCC is a highly aggressive malignancy demanding prompt oncologic intervention, which may include radical surgery, targeted therapy, or immunotherapy, and is associated with a generally poor prognosis (9).

### Red flags against typical XGP

In our patient, four clinical features argued strongly against typical XGP and should have triggered earlier diagnostic escalation: (1) persistently negative urine and blood cultures despite florid pyuria; (2) persistent high-grade fever despite escalation to carbapenem-based therapy; (3) progressive, transfusion-requiring anemia; and (4) minimal interval change on repeat contrast-enhanced CT. These features are atypical for controlled bacterial XGP. Collectively, they define a clinical pattern that should prompt the clinician to reconsider the diagnosis and pursue tissue confirmation rather than continuing to escalate antibiotics.

### CT imaging differentiation

The bear-paw sign is necessary but not sufficient for XGP. This classic CT finding—representing dilated calyces arranged around a central calculus, producing a bear’s claw appearance—is characteristic of XGP and was present in this case. However, its presence does not exclude malignancy. RCC, including sRCC, can coexist with nephrolithiasis and produce perinephric inflammatory changes that mimic XGP (10–12). The bear-paw sign should be interpreted in conjunction with other CT features rather than used in isolation to establish a diagnosis of XGP.

An irregular solid enhancing component is the most important discriminating CT feature. Classical XGP demonstrates dilated calyces filled with lipid-laden debris, producing relatively uniform low-density filling defects without significant internal solid enhancement. The identification of an irregular, solid component with heterogeneous arterial-phase enhancement within an apparent renal inflammatory mass is fundamentally inconsistent with XGP and must trigger consideration of malignancy (2, 13). In the present case, this feature was identifiable on the initial CT (**Figure 1B**) but was not prospectively recognized as a discriminating red flag. Radiologists reporting CT scans in the context of suspected XGP should specifically comment on the presence or absence of irregular solid enhancing components.

Vascular invasion is a malignancy-specific finding. Irregularity or filling defects within the renal vein or inferior vena cava on venous-phase imaging represent evidence of vascular invasion and are essentially absent in XGP. When identified—even subtly, as in this case (**Figure 1F**)—renal vein irregularity should be considered a strong argument for RCC until proven otherwise. Dedicated attention to venous-phase images with multiplanar reconstruction is recommended when evaluating complex renal masses.

The definitive diagnosis was unequivocally established by post-operative histopathological examination and immunohistochemical analysis. Histopathology confirmed the diagnosis of right renal cell carcinoma, sarcomatoid type (sRCC). The tumor was notably large (9.5 cm), exhibited extensive necrosis, and demonstrated aggressive local invasion into the renal capsule, renal pelvis, and perinephric fat. The presence of lymphovascular invasion further underscored the aggressive biological behavior characteristic of sRCC. Definitive diagnosis relies on histopathology. While CD68 positivity is characteristic of the lipid-laden macrophages in XGP, its presence in sRCC (likely due to tumor-infiltrating macrophages) can be confusing (14). However, the strong positivity for PAX-8 and CKpan in our case confirmed the renal epithelial origin of the tumor, effectively excluding primary inflammatory processes. The high Ki-67 index further corroborated the aggressive malignant nature of the lesion.

This case of sarcomatoid renal cell carcinoma masquerading as xanthogranulomatous pyelonephritis illustrates a critical and potentially life-threatening diagnostic pitfall. While overlapping clinical and radiological features between these two entities make early differentiation challenging, a systematic approach to recognizing atypical features—persistently negative cultures, irregular solid enhancing components, imaging evidence of vascular involvement, progressive anemia, and failure to respond to escalating antibiotic therapy—can and should prompt earlier consideration of malignancy. When a presumed renal infection does not behave like an infection, malignancy should be excluded early.

### Critical analysis of the 24-day antibiotic course

We critically acknowledge that the 24-day antibiotic course in this case was excessively prolonged. In retrospect, the constellation of atypical features—negative cultures, persistent fever, progressive anemia, irregular solid enhancement, and suspected vascular invasion—was already evident within the first 7–10 days of admission. Earlier surgical intervention should have been considered at that juncture.

Several alternative strategies could have been employed: (1) repeat contrast-enhanced CT at day 7–10 to assess for interval change, rather than waiting until day 27; (2) ultrasound- or CT-guided percutaneous core needle biopsy of the solid enhancing component, which could have provided tissue diagnosis without the morbidity of nephrectomy; (3) early multidisciplinary consultation to weigh the risks of continued antibiotic therapy against the risks of diagnostic delay. We recognize that earlier diagnosis and surgery may not change the ultimate oncological outcome in advanced sRCC with a Ki-67 index of 50–55%. However, it reduces diagnostic delay, allows timely staging, and enables earlier access to systemic oncological therapy—all of which are important for patient care even when cure is not achievable.

## Conclusion

This case of sarcomatoid renal cell carcinoma masquerading as xanthogranulomatous pyelonephritis illustrates a critical and potentially life-threatening diagnostic pitfall. While overlapping clinical and radiological features between these two entities make early differentiation challenging, a systematic approach to recognizing atypical features—persistently negative cultures, irregular solid enhancing components, imaging evidence of vascular involvement, progressive anemia, and failure to respond to escalating antibiotic therapy—can and should prompt earlier consideration of malignancy. The fundamental clinical principle established by this case is: when a presumed renal infection does not behave like an infection, malignancy should be excluded early.

## Data Availability

The original contributions presented in the study are included in the article/Supplementary Material. Further inquiries can be directed to the corresponding author.
